# Incorporation of high-dose ^131^I-metaiodobenzylguanidine treatment into tandem high-dose chemotherapy and autologous stem cell transplantation for high-risk neuroblastoma: results of the SMC NB-2009 study

**DOI:** 10.1186/s13045-017-0477-0

**Published:** 2017-05-16

**Authors:** Ji Won Lee, Sanghoon Lee, Hee Won Cho, Youngeun Ma, Keon Hee Yoo, Ki Woong Sung, Hong Hoe Koo, Eun Joo Cho, Suk-Koo Lee, Do Hoon Lim

**Affiliations:** 1 Department of Pediatrics, Samsung Medical Center, Sungkyunkwan University School of Medicine, 81 Irwon-ro, Gangnam-gu, Seoul 135-710 Republic of Korea; 2 Department of Pediatric Surgery, Samsung Medical Center, Sungkyunkwan University School of Medicine, 81 Irwon-ro, Gangnam-gu, Seoul 135-710 Republic of Korea; 3 Department of Radiation Oncology, Samsung Medical Center, Sungkyunkwan University School of Medicine, 81 Irwon-ro, Gangnam-gu, Seoul 135-710 Republic of Korea

**Keywords:** Neuroblastoma, High-dose ^131^I-MIBG, High-dose chemotherapy, Autologous stem cell transplantation

## Abstract

**Background:**

In our previous SMC NB-2004 study of patients with high-risk neuroblastomas, which incorporated total-body irradiation (TBI) with second high-dose chemotherapy and autologous stem cell transplantation (HDCT/auto-SCT), the survival rate was encouraging; however, short- and long-term toxicities were significant. In the present SMC NB-2009 study, only TBI was replaced with ^131^I-meta-iodobenzylguanidine (MIBG) treatment in order to reduce toxicities.

**Methods:**

From January 2009 to December 2013, 54 consecutive patients were assigned to receive tandem HDCT/auto-SCT after nine cycles of induction chemotherapy. The CEC (carboplatin + etoposide + cyclophosphamide) regimen and the TM (thiotepa + melphalan) regimen with (for metastatic MIBG avid tumors) or without (for localized or MIBG non-avid tumors) ^131^I-MIBG treatment (18 or 12 mCi/kg) were used for tandem HDCT/auto-SCT. Local radiotherapy, differentiation therapy with 13-cis-retinoic acid, and immunotherapy with interleukin-2 were administered after tandem HDCT/auto-SCT.

**Results:**

Fifty-two patients underwent the first HDCT/auto-SCT and 47 patients completed tandem HDCT/auto-SCT. There was no significant immediate toxicity during the ^131^I-MIBG infusion. Acute toxicities during the tandem HDCT/auto-SCT were less severe in the NB-2009 study than in the NB-2004 study. Late effects such as growth hormone deficiency, cataracts, and glomerulopathy evaluated at 3 years after the second HDCT/auto-SCT were also less significant in the NB-2009 study than in NB-2004 study. There was no difference in the 5-year event-free survival (EFS) between the two studies (67.5 ± 6.7% versus 58.3 ± 6.9%, *P* = 0.340).

**Conclusions:**

Incorporation of high-dose ^131^I-MIBG treatment into tandem HDCT/auto-SCT could reduce short- and long-term toxicities associated with TBI, without jeopardizing the survival rate.

**Trial registration:**

ClinicalTrials.gov NCT03061656

## Background

Although the outcome of high-risk neuroblastoma has improved after the introduction of high-dose chemotherapy and autologous stem cell transplantation (HDCT/auto-SCT), the outcome was still unsatisfactory [1–4]. In recent multicenter clinical trials of HDCT/auto-SCT, the 3- or 5-year event-free survival (EFS) was reported to be 40–50% [5, 6]. The main cause of treatment failure after HDCT/auto-SCT is relapse or tumor progression rather than treatment-related mortality (TRM). In this context, the role of tandem HDCT/auto-SCT has been investigated with the hypothesis that a sequential course of HDCT/auto-SCT might further improve the survival of high-risk neuroblastoma and encouraging survival rates have been reported [7–9]. However, further dose escalation and the addition of more drugs during tandem HDCT/auto-SCT might be associated with more significant short- and long-term toxicities than single HDCT/auto-SCT. Therefore, a strategy to minimize toxicities during or after tandem HDCT/auto-SCT is important for better outcomes.

We previously reported the results of a single-arm prospective trial (SMC NB-2004 study) using tandem HDCT/auto-SCT for high-risk neuroblastoma [10], which was designed based on the results of retrospective analysis of our early experience between the late 1990s and early 2000s [7]. In the NB-2004 study, nine cycles of induction chemotherapy were administered before tandem HDCT/auto-SCT, and total-body irradiation (TBI) was incorporated into the second HDCT/auto-SCT. As a result, survival rates were very encouraging; however, short- and long-term toxicities associated with tandem HDCT/auto-SCT, particularly TBI, were severe.

Around 90% of neuroblastomas express the norepinephrine transporter that facilitates uptake of radiolabeled metaiodobenzylguanidine (MIBG), suggesting a rationale for employing radiolabeled MIBG as a targeted radiotherapy [11, 12]. The safety and efficacy of ^131^I-MIBG treatment has been reported in previous studies that were performed mainly on relapsed or refractory neuroblastoma patients [13–17]. Therefore, we conducted a new prospective trial (SMC NB-2009 study), in which only TBI in the second HDCT/auto-SCT of NB-2004 study was replaced with high-dose ^131^I-MIBG treatment to reduce short- and long-term toxicities, without jeopardizing the survival rate. Otherwise, all treatments including induction treatment, the first HDCT and the post-HDCT/auto-SCT treatment in the NB-2009 study were the same as those in the NB-2004 study.

## Methods

### Patients

From January 2009 to December 2013, patients who were newly diagnosed with high-risk neuroblastoma were enrolled. Patients older than 1 year with International Neuroblastoma Staging System (INSS) stage 4 and patients with MYCN proto-oncogene bHLH transcription factor (*MYCN*) amplified tumors regardless of stage were both categorized as high-risk patients. Tumor extent was evaluated using computed tomography/magnetic resonance imaging, a technetium-99 (^99^Tc) bone scan, bilateral bone marrow examination, an ^123^I-MIBG scan, and ^18^F-fluorodeoxy-d-glucose positron emission tomography (PET)/computed tomography. Tumor staging was determined according to the INSS [18]. The International Neuroblastoma Pathology Classification (INPC) system was used in the pathologic classification of tumors [19], and *MYCN* amplification was determined using interphase fluorescence in situ hybridization on tumor tissues. *MYCN* amplification was defined as a greater than fourfold increase of the *MYCN* signal number relative to the number of control probe signals [20].

### Induction treatment

Induction treatments were the same as those in the SMC NB-2004 study [10]. Briefly, nine cycles of chemotherapy were administered during induction treatment. The CEDC (cisplatin + etoposide + doxorubicin + cyclophosphamide) and ICE (ifosfamide + carboplatin + etoposide) regimens were used alternatively (Table 1). Each cycle of induction chemotherapy was performed at 4-week intervals, and delays were permitted to allow the absolute neutrophil count to recover to 1.0 × 10^9^/L and the platelet count to 100 × 10^9^/L. Surgery was usually performed after six cycles of chemotherapy, except when complete excision was possible at diagnosis. The extent of surgery was resection of the primary tumor along with accessible regional lymph nodes. Peripheral blood stem cells (PBSCs) were collected during the recovery phase of the seventh chemotherapy cycle. The minimum target number of CD34^+^ cells was 2.0 × 10^6^/kg.

**Table 1:** Chemotherapy regimens

Regimen	Drug	Dose	Schedule	Total dose
Induction regimens				
CEDC^a^	Cisplatin	60 mg/m^2^/day	Day 0	60 mg/m^2^
	Etoposide	100 mg/m^2^/day	Days 2, 5	200 mg/m^2^
	Doxorubicin	30 mg/m^2^/day	Day 2	30 mg/m^2^
	Cyclophosphamide	30 mg/kg/day	Days 3, 4	60 mg/kg
ICE^a^	Ifosfamide	1 200 mg/m^2^/day	Days 0–4	6 000 mg/m^2^
	Carboplatin	400 mg/m^2^/day	Days 0, 1	800 mg/m^2^
	Etoposide	100 mg/m^2^/day	Days 0−4	500 mg/m^2^
First HDCT regimen				
CEC	Carboplatin	650 mg/m^2^/day	Days −7, −6, −5	1 950 mg/m^2^
	Etoposide	650 mg/m^2^/day	Days −7, −6, −5	1 950 mg/m^2^
	Cyclophosphamide	1 800 mg/m^2^/day	Days −4, −3, −2	5 400 mg/m^2^
Second HDCT regimens				
^131^I-MIBG-TM	^131^I-MIBG	18 or 12 mCi/kg	Days −21	12 or 18 mCi/kg
	Thiotepa	200 mg/m^2^/day	Days −6, −5, −4	600 mg/m^2^
	Melphalan	60 mg/m^2^/day	Days −3, −2	120 mg/m^2^
TM	Thiotepa	300 mg/m^2^/day	Days −6, −5, −4	900 mg/m^2^
	Melphalan	60 mg/m^2^/day	Days −3, −2	120 mg/m^2^

*MIBG* meta-iodobenzylguanidine

^a^The chemotherapy dose was reduced for patients younger than 24 months of age, i.e., kilogram-based dose for <6 months of age, 70% of dose based on body surface area for 6–11 months of age, 80% of dose for 12–17 months of age, and 90% of dose for 18–23 months of age

### Tandem HDCT/auto-SCT

HDCT regimens were also the same as in the NB-2004 study [10], except that TBI (3.33 Gy/day on days −3, −2, −1) in the second HDCT/auto-SCT was replaced with ^131^I-MIBG treatment in the NB-2009 study (Table 1). CEC (carboplatin + etoposide + cyclophosphamide) and TM (thiotepa + melphalan) with ^131^I-MIBG treatment were used as the first and second HDCT regimen, respectively. During the first year of the study, the dose of ^131^I-MIBG treatment was 18 mCi/kg (maximum 500 mCi), but it was reduced to 12 mCi/kg (maximum 500 mCi) during the remaining study period in fear of late adverse effects of high-dose ^131^I-MIBG treatment. ^131^I-MIBG was infused on day −21 of the second HDCT/auto-SCT. For patients with MIBG non-avid or localized tumors, ^131^I-MIBG treatment was not administered; instead, the dose of thiotepa was increased to 300 mg/m^2^/day. The interval between the first and second HDCT/auto-SCT was set to be 12 ± 1 weeks in order to reduce toxicities in the second HDCT/auto-SCT. However, some delay was allowed when there was a problem in the ^131^I-MIBG supply or scheduling the transplantation unit. Approximately half of the PBSCs collected were infused for bone marrow rescue at each HDCT/auto-SCT session, and the minimum number of CD34^+^ cells was 1.0 × 10^6^/kg.

### Post-HDCT/auto-SCT treatment

Treatments after tandem HDCT/auto-SCT were also the same in the NB-2004 study [10]. Briefly, local radiotherapy (RT) to the site of the primary tumor (15 Gy/1.5 Gy for complete resection of the primary tumor and 21.6 Gy/1.8 Gy or 30.6 Gy/1.8 Gy for incomplete resection according to the residual ^123^I-MIBG or PET uptake) was administered to all patients approximately 6 weeks after the second HDCT/auto-SCT. RT was not administered to the residual metastatic sites. Differentiation therapy with 13-*cis*-retinoic acid (125 mg/m^2^/day for 14 days every 4 weeks) and immunotherapy with IL-2 (2 × 10^6^ U/m^2^/day, subcutaneous injection for five consecutive days every 4 weeks) were started at approximately 9 weeks after the second HDCT/auto-SCT and continued until a year after the second HDCT/auto-SCT.

### Response criteria

Response was evaluated every three cycles of induction chemotherapy, before the first and second HDCT/auto-SCT, every 3 months for the first year after the second HDCT/auto-SCT, every 4 months for the second year, every 6 months for the third year, and every year thereafter. Basically, International response criteria for neuroblastoma were used to evaluate the treatment response [18]. CT/MRI, ^99^Tc- bone scan, ^123^I-MIBG scan, and bilateral bone marrow examinations were routinely performed to determine the response at primary and metastatic sites. The results of the ^123^I-MIBG scan and bone marrow examination were also integrated into the response assessment of metastatic sites according to the international response criteria. Briefly, a complete response (CR) was defined as no identifiable tumor with normal catecholamine. A very good partial response (VGPR) was defined as a reduction of the primary tumor by 90–99% with normal catecholamine with or without any residual ^99^Tc bone changes. A partial response (PR) was defined as a reduction of the primary tumor and metastatic tumor by >50%. A mixed response (MR) was defined as a reduction of any measurable lesion by >50% with a reduction of any other lesion by <50%. Stable disease (SD) was defined as no new lesion and an increase in any existing lesion by <25%. Progressive disease (PD) was defined as any new lesion or increase of any measurable lesion by >25%.

### Evaluation of late adverse effects

Cardiac, renal, respiratory, endocrine, auditory, and ophthalmologic functions were evaluated 1, 3, and 5 years after the second HDCT/auto-SCT. Respiratory function was assessed by clinical signs and symptoms in all patients, and a pulmonary function test was performed if the patient could cooperate with the test. Neurocognitive functions were evaluated 3 years after the second HDCT/auto-SCT using the Korean-Wechsler Preschool and Primary Scale of Intelligence (<6 years of age) or the Korean version of the Wechsler Intelligence Scale for children-III (≥6 years of age). Toxicities were recorded according to the Common Terminology Criteria for Adverse Events (version 4.0) of the US National Cancer Institute.

### Survival analysis and statistics

Differences in categorical variables were measured by the chi-square test or Fisher’s exact test, and differences in continuous variables were calculated with the Mann-Whitney *U* test. EFS was calculated from the date of diagnosis until the date of relapse, progression, secondary malignancy, or death, whichever occurred first. Overall survival (OS) was calculated from the date of diagnosis until death from any cause. Survival rates and standard errors were estimated using the Kaplan-Meier method. Differences in survival rates between the two groups were compared using the log-rank test. *P* values <0.05 were considered significant.

## Results

### Patient characteristics

A total of 54 consecutive patients were enrolled, and the patient’s characteristics are summarized in Table 2. The median age at diagnosis was 30.5 (range, 1–231) months, and 45 (83.3%) of patients were older than 18 months. Fifty-two (96.3%) patients had metastatic tumors and 25 (48.1%) patients had *MYCN*-amplified tumors. While two patients with stage 4, *MYCN* non-amplified tumors in the NB-2004 study who were aged between 12 and 18 months were not classified in the high-risk group according to the International Neuroblastoma Risk Group classification [21], there was no such patient in the NB-2009 study. Otherwise, there was no significant difference in the clinical and biological characteristics of the patients between the NB-2004 and NB-2009 studies.

**Table 2:** Patient characteristics

Parameters	NB-2004 (*n* = 50)	NB-2009 (*n* = 54)	*P* value
Age (months)	37 (4–129)	30.5 (1–231)	0.460
Age >18 months, *n* (%)	42 (84.0)	45 (83.3)	0.927
INSS stage, *n* (%)			0.116
Stage 3	5 (10.0)	2 (3.7)	
Stage 4	45 (90.0)	49 (90.7)	
Stage 4S	0 (0.0)	3 (5.6)	
*MYCN* amplification, *n* (%)	22 (44.0)	25 (48.1)	0.680
Unfavorable pathology (INPC), *n* (%)	37/43 (86.0)	39/52 (75.0)	0.180
Serum LDH (IU/L), median (range)	1766 (505–15 720)	1746 (416–14 435)	0.542
Serum ferritin (ng/mL), median (range)	226 (20–3 284)	266 (25–1491)	0.972
Serum NSE (ng/mL), median (range)	141.2 (17.9–1 507.1)	97.6 (10.3–1 815.0)	0.134
24-h urine VMA (mg), median (range)	15.0 (0.7–100.0)	9.3 (0.4–92.4)	0.576

*Abbreviations*: *INSS* International Neuroblastoma Staging System, *INPC* International Neuroblastoma Pathology Classification, *LDH* lactate dehydrogenase, *NSE* neuron-specific enolase, *VMA* vanillylmandelic acid

### Induction treatment

Neutropenic fever was experienced by all patients during induction treatment; however, non-hematologic toxicity was not significant (Table 3). Surgery was performed before initiation of induction chemotherapy in eight patients; however, in 42 patients, it was deferred until after six (*n* = 40) or more (*n* = 2) cycles of induction chemotherapy. In the remaining four patients, surgery was not performed because the tumor disappeared during induction treatment (*n* = 1), was still unresectable after induction treatment (*n* = 2), or progressed before surgery (*n* = 1). Two patients experienced PD during induction treatment and died despite salvage treatment.

**Table 3:** Grade 3/4 toxicities during induction chemotherapy

Parameter	CEDC (261 cycles)	ICE (210 cycles)
Hematologic toxicity		
Duration of neutropenia, day (range)	6 (0–19)	9 (0–25)
No. of platelet transfusions, *n* (range)	2 (0–27)	3 (0–15)
Neutropenic fever, *n* (%)	71 (27.2)	94 (44.8)
Positive blood culture, *n* (%)	4 (1.5)	12 (5.7)
Non-hematologic toxicity		
Elevation of liver enzymes, *n* (%)	10 (3.8)	16 (7.6)
Hyperbilirubinemia, *n* (%)	1 (0.4)	0 (0.0)
Renal insufficiency, *n* (%)	0 (0.0)	0 (0.0)
Hyponatremia, *n* (%)	7 (2.9)	4 (1.9)
Hypokalemia, *n* (%)	15 (5.7)	13 (6.2)

*Abbreviations*: *CEDC* cisplatin + etoposide + doxorubicin + cyclophosphamide, *ICE* ifosfamide + carboplatin + etoposide

### Tandem HDCT/auto-SCT

Fifty-two patients proceeded to the first HDCT/auto-SCT, and their tumor statuses at the first HDCT/auto-SCT were CR in 29 (53.7%), VGPR in 10 (18.5%), PR in 11 (20.4%), MR in one (1.9%), and SD in one (1.9%).

**Fig. 1:**
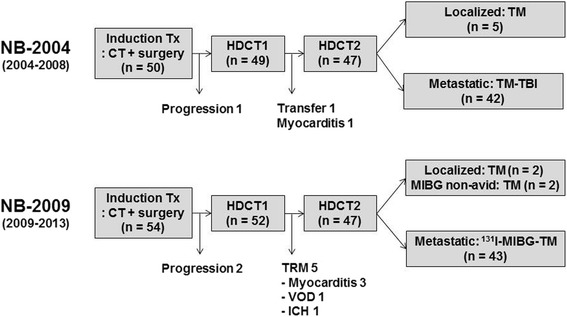
Treatment flow. Treatment flow of the patients is illustrated. *CT* chemotherapy, *HDCT1* first high-dose chemotherapy, *HDCT2* second HDCT, *TBI* total-body irradiation, *TRM* treatment-related mortality, *Tx* treatment, *VOD* hepatic veno-occlusive disease, *ICH* intracranial hemorrhage

Figure 1 shows the patient flow. Five of 52 patients who underwent the first HDCT/auto-SCT died from TRM (myocarditis in three, hepatic veno-occlusive disease in one, and intracranial hemorrhage in one) during the first HDCT/auto-SCT. Therefore, 47 patients could proceed to the second HDCT/auto-SCT. Forty-three patients received ^131^I-MIBG treatment (18 mCi/kg in five patients and 12 mCi/kg in 38 patients), and the remaining four patients with a localized tumor (*n* = 2) or a MIBG non-avid tumor (*n* = 2) underwent the second HDCT/auto-SCT without ^131^I-MIBG treatment. The median interval from day 0 of the first HDCT/auto-SCT to initiation of the second HDCT/auto-SCT was 87 days (range 79–139). The interval was within 12 ± 1 weeks in 36 (76.6%) patients and was less than 14 weeks in 45 (95.7%) patients.

Table 4 lists hematologic and non-hematologic toxicities of grade ≥3 that developed within 1 month of each HDCT/auto-SCT. Hematologic recovery was rapid in both the first and second HDCT/auto-SCT, although platelet recovery was delayed in the second HDCT/auto-SCT compared to the first (*P* = 0.004). The grade ≥3 non-hematologic toxicities that developed in more than 30% of patients were diarrhea, an elevation of liver enzymes and hypokalemia in the first HDCT/auto-SCT, and stomatitis and diarrhea in the second HDCT/auto-SCT. No acute complications occurred during or shortly after the infusion of ^131^I-MIBG. In the comparison of toxicities during the second HDCT/auto-SCT between the TM-TBI regimen in the NB-2004 study (*n* = 42) and the ^131^I-MIBG-TM regimen in the NB-2009 study (*n* = 43), hematologic recovery was faster and duration of fever was shorter in the NB-2009 study than in the NB-2004 study (*P* = 0.038 for ANC recovery and *P* = 0.041 for platelet recovery). In addition, the frequency of stomatitis, elevation of liver enzymes, and hypokalemia was lower in the NB-2009 study than in the NB-2004 study (*P* = 0.003, 0.030, and <0.001, respectively). Acute toxicities that occurred more than 1 month after HDCT/auto-SCT were cytomegalovirus disease in two patients and *Pneumocystis jirovecii* pneumonia in one patient, but they all improved after treatment.

**Table 4:** Comparison of toxicities grade ≥ 3 during tandem HDCT/auto-SCT

	First HDCT in NB-2009 (*n* = 52)	Second HDCT in NB-2009 (*n* = 47)	*P* value	TM-TBI in NB-2004 (*n* = 42)	^131^I-MIBG-TM in NB-2009 (*n* = 43)	*P* value
Hematologic toxicity						
CD34^+^ cells (×10^6^/kg), median (range)	2.7 (1.0–14.1)	2.4 (1.0–14.1)	0.321	1.9 (1.0–16.0)	2.3 (1.0–14.1)	0.184
Time (days) to reach an ANC 0.5 × 10^9^/L^a^, median (range)	11 (9–14)	10 (8–15)	0.339	11 (8–16)	10 (8–15)	0.038
Time (days) to reach a PLT count 20 × 10^9^/L^b^, median (range)	24 (13–52)	32 (16–275)	0.004	40 (18–1 248)	32 (16–275)	0.041
Days of BT ≥38.0 °C, median (range)	3 (0–16)	4 (0–11)	0.221	8 (2–27)	4 (0–11)	<0.001
Positive blood culture, *n* (%)	3 (5.8)	3 (6.4)	0.898	2 (4.8)	3 (7.0)	0.511
Non-hematologic toxicity						
Vomiting, *n* (%)	12 (23.1)	6 (12.8)	0.184	11 (26.2)	5 (11.6)	0.086
Stomatitis, *n* (%)	9 (17.3)	18 (38.3)	0.019	28 (66.7)	15 (34.9)	0.003
Diarrhea (frequency ≥10/day), *n* (%)	16 (30.8)	17 (36.2)	0.569	23 (54.8)	15 (34.9)	0.065
Elevation of liver enzyme, *n* (%)	44 (84.6)	1 (2.1)	<0.001	7 (16.7)	1 (2.3)	0.030
Hepatic VOD, *n* (%)	3 (5.8)	0 (0.0)	0.245	4 (9.5)	1 (2.3)	0.202
Renal insufficiency, *n* (%)	4 (7.7)	0 (0.0)	0.119	2 (4.8)	0 (0.0)	0.241
Hypokalemia, *n* (%)	17 (32.7)	3 (6.4)	0.001	17 (40.5)	3 (7.0)	<0.001
Myocarditis, *n* (%)	3 (5.8)	0 (0.0)	0.245	0 (0.0)	0 (0.0)	>0.999
Intracranial hemorrhage, *n* (%)	1 (1.9)	0 (0.0)	0.525	0 (0.0)	0 (0.0)	>0.999
Toxic death, *n* (%)	5 (9.6)	0 (0.0)	0.058	0 (0.0)	0 (0.0)	>0.999
Time (days) to discharge from day 0 of SCT, median (range)	17 (12–51)	18 (12–31)	0.135	19 (15–81)	18 (12–31)	0.098

*Abbreviations*: HDCT/auto-SCT high-dose chemotherapy and autologous stem cell transplantation, *ANC* absolute neutrophil count, *PLT* platelet, *BT* body temperature, *VOD* veno-occlusive disease

^a^The first day when ANC exceeded 0.5 × 10^9^/L for three consecutive days

^b^The first day when PLT count exceeded 20 × 10^9^/L without transfusion for 7 days

### Relapse and survival

Tumor relapse or progression occurred in 15 (27.8%) patients (two during induction treatment and 13 after the second HDCT/auto-SCT), and eight (14.8%) of them died despite salvage treatment. All 13 relapse/progressions after tandem HDCT/auto-SCT occurred at metastatic sites (bone in seven, brain/dura in five, bone marrow in four, and lymph node in two) with (*n* = 2) or without (*n* = 11) primary site relapse. Seven patients experienced relapse at multiple sites, and six patients experienced relapse at a single site.

**Fig. 2:**
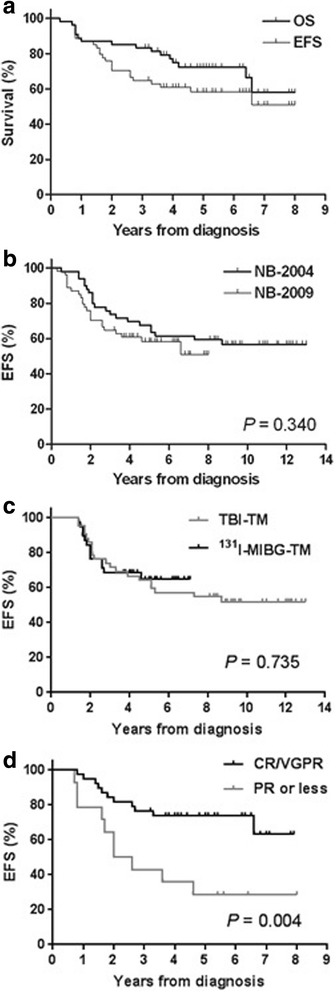
Survival rates. The 5-year OS and EFS rates after diagnosis were 72.4 ± 6.4% and 58.3 ± 6.9%, respectively (**a**). There was no difference in EFS between the NB-2004 and NB-2009 studies (**b**). There was no difference in EFS between the TM-TBI regimen in the NB-2004 study and 12 mCi/kg of ^131^I-MIBG-TM in the NB-2009 study (**c**). EFS of patients in CR or VGPR at the first HDCT/auto-SCT was higher than in those patients with PR or worse (**d**)

TRM occurred in eight (14.8%) patients: five (9.3%) from acute toxicities, two (3.7%) from chronic lung disease, and one (1.9%) from secondary malignancy. Five patients died from acute toxicities during the first HDCT/auto-SCT as described above. Two of five patients who received 18 mCi/kg of ^131^I-MIBG treatment died from chronic lung disease at 40 and 79 months from diagnosis, respectively. These two patients were in a CR prior to tandem HDCT/auto-SCT. One patient was diagnosed with myelodysplastic syndrome at 19 months after diagnosis and died. Therefore, 31 out of 54 patients remained event-free with a median follow-up of 65 months (range, 38–96) from diagnosis, and 38 of 54 patients remained alive with a median follow-up of 63 months (range, 38–96) from diagnosis. The 5-year OS and EFS rates after diagnosis were 72.4 ± 6.4% and 58.3 ± 6.9%, respectively (Fig. 2a). There was no difference in EFS between the NB-2004 and NB-2009 studies (*P* = 0.340, Fig. 2b). When the analysis was confined only to 38 patients with metastatic MIBG avid tumors who received 12 mCi/kg of ^131^I-MIBG treatments, survival rates were not different from those in the 42 patients who underwent the second HDCT/auto-SCT with the TM-TBI regimen in the NB-2004 study (*P* = 0.735, Fig. 2c). The EFS rates of patients with CR or VGPR at the first HDCT/auto-SCT were higher than that of patients with PR or worse (5-year EFS, 73.6 ± 7.2% versus 25.0 ± 10.8%, *P* = 0.004, Fig. 2d).

### Late adverse effects

All 27 patients who remained event-free 3 years after the second HDCT/auto-SCT were evaluated for the presence of various late adverse effects (Table 5). Twenty-three patients had at least 1, 18 had ≥2, and eight had ≥3 late adverse effects. Sensory neural hearing loss, renal tubulopathy, and hypothyroidism were common late adverse effects. Among the 27 patients, 16 patients underwent a pulmonary function test for screening for chronic lung disease. The FEV1 of the patients ranged from 70 to 132% (median 91%) of the expected normal value, and grade 3/4 FEV1 reduction was not observed in any patients. All late adverse effects were grades 1 or 2 except in two patients with sensorineural hearing loss requiring hearing aids. The frequency of growth hormone deficiency, cataracts, and renal glomerulopathy was lower in the NB-2009 study than in the NB-2004 study. The median number of late effects in each patient was 2 (range 0–4), and it was lower than that in the NB-2004 study.

**Table 5:** Late adverse effects at 3 years after tandem HDCT/auto-SCT

	NB-2004 (*n* = 32)	NB-2009 (*n* = 27)	*P* value
Endocrinopathy			
Hypothyroidism, *n* (%)	12 (37.5)	11 (40.7)	0.799
Growth hormone deficiency, *n* (%)	9 (28.1)	1 (3.7)	0.016
Glucocorticoid deficiency, *n* (%)	1 (3.1)	0 (0.0)	0.542
Sex hormone deficiency, *n* (%)	1/2^a^ (50.0)	1/3^a^ (33.3)	0.700
Height, *Z*-score, median (range)	−1.46 (−5.66–0.93)	−0.65 (−2.78–1.40)	0.004
Weight, *Z*-score, median (range)	−0.84 (−2.91–0.75)	−0.32 (−2.15–2.08)	0.014
BMI, *Z*-score, median (range)	−0.38 (−2.58–1.62)	−0.42 (−1.86–2.68)	0.166
Hearing loss, *n* (%)	24 (75.0)	16 (59.3)	0.197
Cataract, *n* (%)	9 (28.1)	0 (0)	0.002
Chronic lung disease, *n* (%)	2 (6.3)	0 (0.0)	0.673
Renal			
Glomerulopathy, *n* (%)	18 (56.3)	4 (14.8)	0.001
Tubulopathy, *n* (%)	20 (62.5)	17 (63.0)	0.971
Cardiac dysfunction, *n* (%)	0 (0.0)	0 (0.0)	1.000
Presence of grade 3 dysfunction, *n* (%)	4 (12.5)	2 (7.4)	0.678
Number of dysfunctions/patient, median (range)	3 (0–7)	2 (0–4)	0.003

*BMI* body mass index

^a^Only adolescents were analyzed

**Fig. 3:**
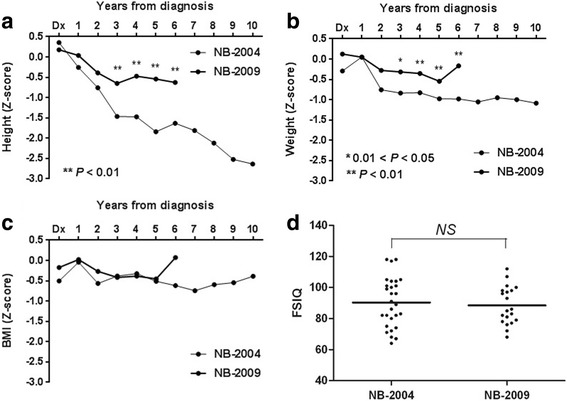
Long-term sequelae after tandem HDCT. Vertical growth retardation (**a**) and poor weight gain (**b**) was less significant in the NB-2009 study compared to the NB-2004 study. Body mass index was not different between the two studies (**c**). There was no difference in full-scale intelligence quotient (FSIQ) between the NB-2004 and NB-2009 studies (**d**)

Vertical growth retardation and poor weight gain were less significant in the NB-2009 study than in the NB-2004 study (Fig. 3a, b). Body mass index was not different between the two studies (Fig. 3c). The full-scale intelligence quotient was 86 (range 57–112), and there was no difference between the NB-2004 and NB-2009 studies (Fig. 3d).

## Discussion

Our previous prospective study (NB-2004 study), which incorporated TBI into the second HDCT/auto-SCT, showed very encouraging survival rates. However, we found that late adverse effects, particularly from TBI, occurred in all long-term survivors. Therefore, we conducted a new prospective trial, which incorporated high-dose ^131^I-MIBG treatments into the second HDCT/auto-SCT instead of TBI. The survival rates were comparable to those in the NB-2004 study; however, the short- and long-term toxicities were significantly less than in the NB-2009 study.

Several phase I and II clinical trials using ^131^I-MIBG treatment were conducted in patients with relapsed or refractory neuroblastoma, and the response rates were 30–60% [13–17]. More recently, studies incorporating ^131^I-MIBG treatments as part of myeloablative therapy have been reported [22–24]. However, no studies to date have incorporated high-dose ^131^I-MIBG treatments into tandem HDCT/auto-SCT for treating newly diagnosed high-risk patients. To the best of our knowledge, the NB-2009 study is the first prospective study that incorporated high-dose ^131^I-MIBG treatments into tandem HDCT/auto-SCT as a front-line treatment in a substantial number of patients.

Early clinical trials showed that myelosuppression was the main dose-limiting toxicity of ^131^I-MIBG treatment. Matthay et al. reported that the maximum-tolerated ^131^I-MIBG dose was 12 mCi/kg for patients without available stem cells, while 18 mCi/kg was tolerable and feasible for patients with available stem cells [16]. The New Approaches to Neuroblastoma Therapy (NANT) consortium conducted a phase I dose escalation study to determine the maximum-tolerated dose and toxicity of ^131^I-MIBG treatment incorporated into the CEM (carboplatin + etoposide + melphalan) regimen in refractory neuroblastoma patients [23]. The results of the NANT study suggested that the maximal tolerable dose was 12 mCi/kg of ^131^I-MIBG in the normal renal function cohort. In another study, ^131^I-MIBG at doses up to 18 mCi/kg could be safely administered 6 weeks prior to a BM (busulfan + melphalan) regimen for refractory neuroblastoma [22]. In the present NB-2009 study, the dose of ^131^I-MIBG treatment was 18 mCi/kg in the first year of the study, but it was reduced to 12 mCi/kg in the remaining study period out of concern about late adverse effects of ^131^I-MIBG treatment coupled with tandem HDCT/auto-SCT. Short-term toxicities during the second HDCT/auto-SCT were acceptable. As compared to findings in the NB-2004 study, hematologic recovery was faster and fever duration was shorter. In addition, acute non-hematologic toxicities were tolerable, although mucositis-related toxicities were severe. The frequency of stomatitis, elevation of liver enzymes, and hypokalemia were lower than those in the NB-2004 study. No toxic death occurred during the second HDCT/auto-SCT.

Late adverse effects of the ^131^I-MIBG treatment were also reported in several studies. Hypothyroidism has been reported in patients who were treated with ^131^I-MIBG [25, 26]. A secondary malignancy has also been reported after ^131^I-MIBG treatment [27]. However, the long-term adverse effects could not be evaluated properly because ^131^I-MIBG treatments were usually used as salvage treatments for patients with relapsed or refractory tumors, and therefore, sufficient follow-up was not possible in most patients. Late adverse effects in the NB-2009 study evaluated at 3 years after tandem HDCT/auto-SCT were generally milder than those in the NB-2004 study. However, two of five patients who received 18 mCi/kg of ^131^I-MIBG treatment during the first year of the study died from chronic lung disease while there was no death from late toxicities in the 38 patients who received 12 mCi/kg of ^131^I-MIBG treatment during the remaining study. These findings suggest that 18 mCi/kg of ^131^I-MIBG treatment might be beyond the maximal tolerable dose when it is coupled with tandem HDCT/auto-SCT.

Survival rates in the NB-2009 study were encouraging and were not different from those in the NB-2004 study. However, many patients still experienced tumor relapse or progression after the tandem HDCT/auto-SCT, particularly poor responders following induction therapy. There was no difference in clinical and biologic factors between the good and poor responders to induction treatment. All relapsed patients experienced metastatic relapse, and these finding suggest that further improvements in treatment need to be focused on systemic tumor control for high-risk neuroblastoma patients. Anti-GD2 treatment after tandem HDCT/auto-SCT could be one of the options to further improve the outcome for high-risk neuroblastoma patients [28–33]. In a recent phase III randomized clinical trial of tandem versus single HDCT/auto-SCT by the Children’s Oncology Group, the 3-year EFS was reported to be 73.7% in patients who received tandem HDCT/auto-SCT and post-consolidative immunotherapy [34]. Cellular immunotherapy such as adoptive transfer of chimeric antigen receptors (CAR) of T cells or NK cells could also be considered [35–38]. A combination of these immunotherapies following tandem HDCT/auto-SCT might further improve outcomes of high-risk neuroblastoma.

Five patients died from acute toxicities during the first HDCT/auto-SCT, and the tumor status before the first HDCT/auto-SCT was CR or VGPR in three of the five patients. In addition, the two patients who died from chronic lung disease after 18 mCi/kg of ^131^I-MIBG treatment were in a CR before the first HDCT/auto-SCT. This TRM rate is higher than in other studies. In this study, the dose of the first HDCT was same in all patients; however, the intensity of the first HDCT might be relatively higher in patients who are susceptible to the toxicity of chemotherapy, particularly in good responders to induction treatment. Similarly, the toxicity of ^131^I-MIBG treatment might be relatively higher in good responders than in poor responders.

In our previous study evaluating the relationship between an early response during induction treatment and subsequent outcome in high-risk neuroblastoma, relapse-free survival was higher in good responders than in poor responders; however, the TRM rate during tandem HDCT/auto-SCT was also relatively higher in good responders [39]. Therefore, it may be necessary to reduce the treatment intensity to reduce the TRM in the good responders. To the contrary, it might also be necessary to intensify the treatment, such as an increase of the ^131^I-MIBG dose, in the poor responders to reduce the relapse rate despite a possible increase in acute and chronic toxicities. Tailoring the HDCT intensity based on the response during induction treatment might improve the survival rate by minimizing the TRM and relapse rates.

## Conclusions

In summary, incorporation of high-dose ^131^I-MIBG treatment into tandem HDCT/auto-SCT was feasible and could reduce short- and long-term toxicities without jeopardizing survival rates. However, this study is a single-arm prospective study of a relatively small number of patients; therefore, a randomized prospective study with a larger number of patients is needed to confirm the findings of our study. In addition, further improvements in treatment are still needed in order to improve patient outcomes. Combinations of immunotherapeutics and/or tailored therapy according to prior tumor response are possible options needing further investigation.

## Abbreviations

^99^Tc: Technetium-99
auto-SCT: Autologous stem cell transplantation
BM: Busulfan + melphalan
CAR: Chimeric antigen receptors
CEC: Carboplatin + etoposide + cyclophosphamide
CEDC: Cisplatin + etoposide + doxorubicin + cyclophosphamide
CEM: Carboplatin + etoposide + melphalan
CR: Complete response
EFS: Event-free survival
HDCT: High-dose chemotherapy
ICE: Ifosfamide + carboplatin + etoposide
INPC: International Neuroblastoma Pathology Classification
INSS: International Neuroblastoma Staging System
MIBG: Metaiodobenzylguanidine
MR: Mixed response
MYCN: MYCN proto-oncogene, bHLH transcription factor
NANT: New Approaches to Neuroblastoma Therapy
OS: Overall survival
PBSCs: Peripheral blood stem cells
PD: Progressive disease
PR: Partial response
RT: Radiotherapy
SD: Stable disease
TBI: Total-body irradiation
TM: Thiotepa + melphalan
TRM: Treatment-related mortality
VGPR: Very good partial response
